# Clinical management and outcomes of cesarean section in three black-tufted marmosets (*Callithrix penicillata*) —A case series

**DOI:** 10.3389/fvets.2026.1857325

**Published:** 2026-07-02

**Authors:** Anand Rajshekhar Dadke, Prashant Shivaji Gurav, Mahesh Babasaheb Pawar, Swapnil Ashokrao Jadhav, Rucha Gaurav Shrivastav, A Ramakrishnan

**Affiliations:** Department of Wildlife Health and Management, Greens Zoological Rescue and Rehabilitation Centre, Jamnagar, Gujarat, India

**Keywords:** black-tufted marmoset, *Callithrix penicillata*, cesarean section, dystocia, new world monkeys

## Abstract

Black-tufted marmoset (*Callithrix penicillata*) is a New World primate species native to the Brazilian Central Plateau. Over a four-month period, three unrelated adult females presented with prolonged and difficult labor lasting 6–8 h that began during the midnight hours. Radiographic examination in ventrodorsal and lateral views revealed the presence of twin fetuses in all three marmosets. Ultrasonographic evaluation confirmed the viability of all fetuses. Due to the small body size of the species, obstetrical manipulations were considered impractical and therefore not attempted. Surgical intervention was performed under gas anesthesia (isoflurane). With the animals positioned in dorsal recumbency, a 5 cm straight incision was made along the midline through the linea alba to access the abdominal cavity. Fetuses were successfully retrieved and uterus was subsequently closed using an inversion suture pattern, followed by apposition of the muscle layers with polyglactin 910 in an opposition suture pattern, providing effective anatomical closure. The females recovered uneventfully from anesthesia, and complete healing of the surgical wound was observed within 14 days during postoperative follow-up. All six newborn babies were successfully hand-reared. Biometric measurements of newborns were recorded.

## Introduction

The family Callitrichidae includes New World Monkeys such as marmosets and tamarins. The black-tufted marmoset (*Callithrix penicillata*) is characterized by distinctive black tufts of hair surrounding the ears, a long tail with alternating black and white rings and is native to regions extending from the Atlantic coastal forests to the central Caatinga of Brazil (1). The gestation period in marmosets typically ranges from 140 to 150 days, and adults weigh approximately 350 g (2).

Female callitrichids show considerable variation in ovulation patterns, with the number of ovulating follicles ranging from one to four, which corresponds to similarly variable litter sizes (3). Marmosets possess a hemochorial placenta, and littermates typically share a single placental mass and circulation (4). Obstetric interventions in these primates are rarely reported, largely because they are infrequently presented in clinical settings and reproductive complications requiring veterinary assistance appear to occur relatively infrequently (5). Common reproductive procedures performed in marmosets include cesarean section, oocyte retrieval, ovariectomy, and vasectomy (6).

In non-human primates of the genus Callithrix, giving birth to multiple offspring is a common reproductive pattern. Twins are the most typical outcome, although single births or triplets can occasionally occur (5). Marmosets kept in captivity can experience a range of reproductive complications, with dystocia being one of the more commonly observed issues (7). Dystocia is most commonly seen in first-time mothers or in females carrying a single, unusually large fetus. However, it can also occur in those carrying twins, triplets, or even quadruplets, particularly if the newborns become entangled during delivery (8).

## Case description and history

Black-tufted marmosets were housed at the Radha Krishna Temple Elephant Welfare Trust, Jamnagar, Gujarat, India and study was conducted over a four-month period. At the time of acquisition, the animals were already confirmed to be pregnant. They were maintained in enclosures that provided both indoor and outdoor access. The animals were fed four times daily, and their diet consisted of fruits, acacia gum, rice mixed with eggs, live insects (mealworms, superworms, and crickets), mixed vegetables, and commercially available primate pellets.

All three adult females, weighing 400 gms, 430 gms, and 450 gms were confirmed to be in advanced stages of pregnancy by radiography, physical examination and signs of parturition. In diurnal non-human primates, normal parturition typically occurs between 1,900 h and 0800 h (9). Caregivers observed signs including abdominal distension, dullness, tachypnea, vaginal discharge, and repeated straining. Previous reports indicate that the expulsion phase of parturition in marmosets is usually rapid (10). Given these observations and inability to deliver naturally the animals were transferred to the veterinary hospital for further clinical evaluation after close observation.

The three animals presented with the following signs of dystocia:

Labor commenced around midnight in all animals characterized by intense abdominal straining, presence of blood-tinged discharge on the enclosure floor, and a moist perivulvar region. The animals remained active and alert; therefore, initial management consisted of close observation. However, due to failure of progression of parturition by the following morning surgical intervention was indicated, and a cesarean section was performed. Summary of clinical characteristics, anesthetic management and outcomes of the three marmosets undergoing cesarean section are described in Table 1.

**Table 1:** Summary of clinical characteristics, anesthetic duration and outcomes of the three marmosets undergoing cesarean section.

Animal details	Maternal weight (g)	Fetal presentation	Duration of labor before intervention (h)	Anesthetic duration (min)	Maternal outcome	Neonatal outcome
Animal 1	400	Posterior	6	40	Survived; recovered smoothly	Live twin neonates: both survived
Animal 2	430	Anterior	7	35	Survived; recovered uneventfully	Live twin neonates: both survived
Animal 3	450	Posterior	7	30	Survived; recovered smoothly	Live twin neonates: both survived

### Clinical evaluation and diagnosis

A thorough physical and clinical examination was performed in all animals. Vital parameters were within normal physiological limits at the time of evaluation. Blood samples (1 ml) were collected from the femoral vein for routine hematological and biochemical analysis in anticipation of a possible cesarean section. The results of all vital parameters were within normal reference ranges (Table 2).

**Table 2:** Hematobiochemical parameters of marmosets.

Parameter	Unit	Animal 1	Animal 2	Animal 3
White blood cells	10^3^/mm^3^	6.6	7.3	5.1
Lym %	%	37	43	62
Mon %	%	01	03	1
Gra %	%	61	54	36
Eos %	%	1	1	1
Red blood cells	10^6^/mm^3^	9.06	7.94	6.86
Haemoglobin	g/dl	17.3	17.2	16
Haematocrit	%	55.9	56.9	50.8
Mean corpuscular volume	um^3^	62	72	74
Mean concentration of hemoglobin	pg	19.1	21.6	23.3
Mean corpuscular hemoglobin concentration	g/dl	31	30.2	31.5
Platelets	10^5^/mm^3^	623	674	485
SGOT	mg/dl	50	63	112
SGPT	mg/dl	12	8	15
Alkaline phosphatase	mg/dl	268	109	292
Total protein	mg/dl	7.1	6.9	6.9
Albumin	mg/dl	5	5.4	5.6
Total bilirubin	mg/dl	0.2	0.7	0.1
Direct bilirubin	mg/dl	0.1	0.2	0.1
Blood urea nitrogen	mg/dl	11.7	10.8	6.6
Creatinine	mg/dl	0.19	0.21	0.28
Calcium	mg/dl	9.5	9.2	9.9
Inorganic phosphorus	mg/dl	3.5	5.8	4.5
Sodium	mEq/l	150	150	155
Potassium	mEq/l	2.9	5.5	4
Chloride	mEq/l	108	108	110

**Figure 1:**
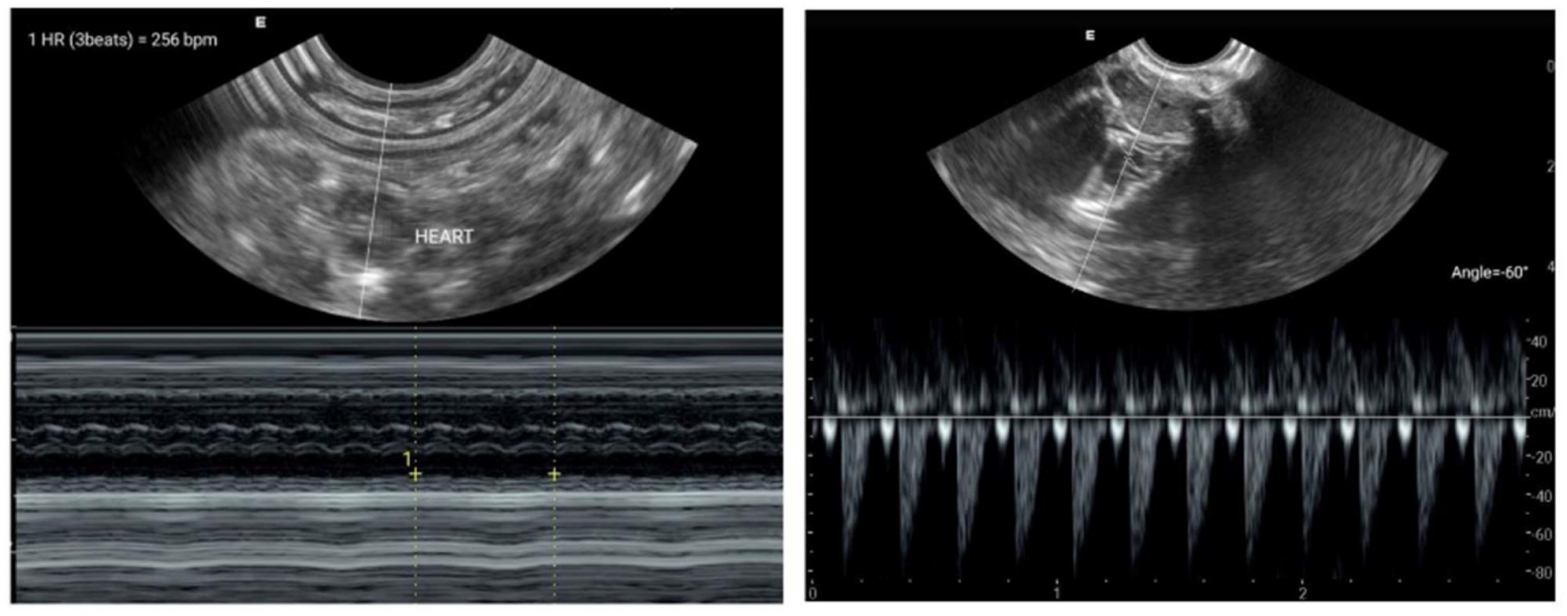
Prenatal ultrasonographic assessment of fetal viability in a black-tufted marmoset.

**Figure 2:**
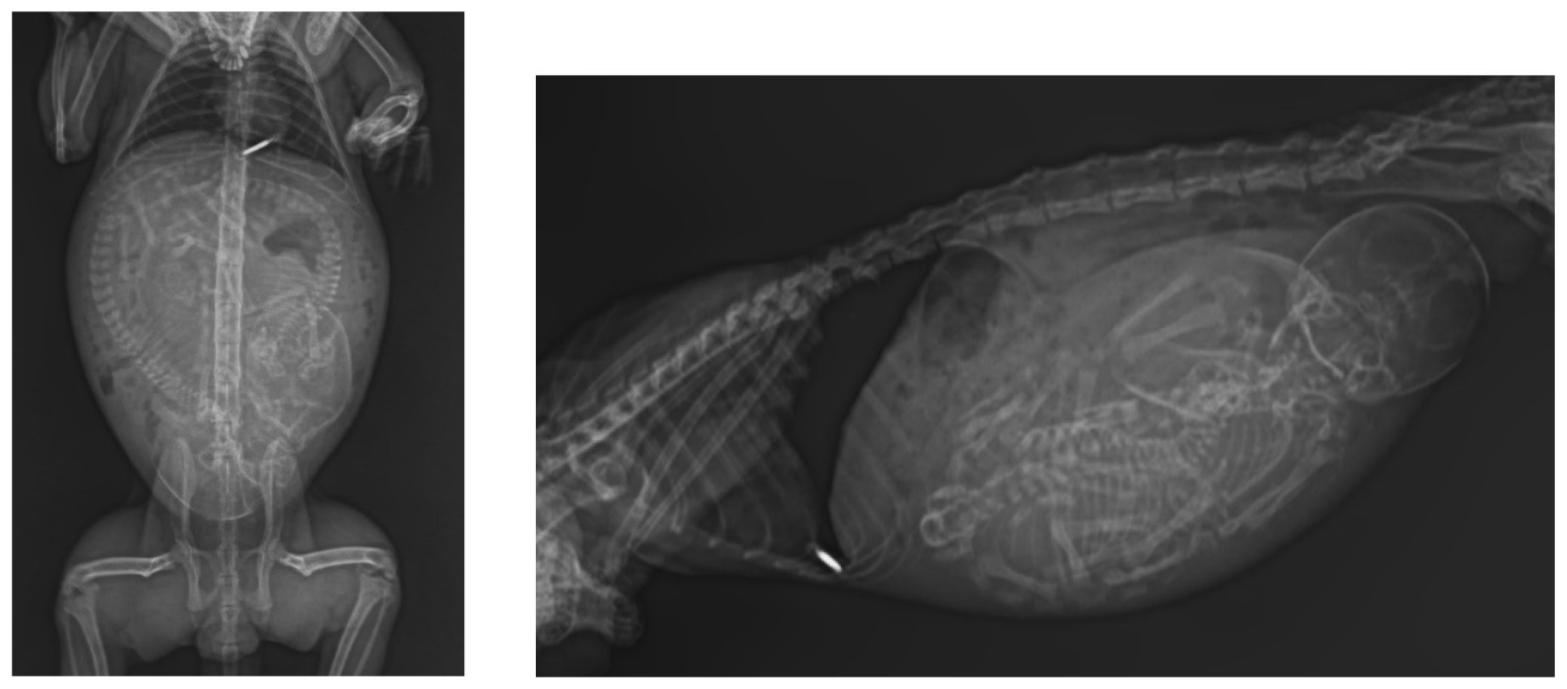
Ventro-dorsal and lateral radiographic projection used for prenatal assessment in a black-tufted marmoset.

Ultrasonography performed with a GE Vivid iQ™ ultrasound system and an 8C-RS micro convex transducer (4–10 MHz) confirmed the presence of viable fetuses in each of the three cases (Figure 1). Radiographic imaging using a MIKASA TRB9020V^TM^ system in conjunction with a Veterinary Digital Radiography Operator Console (VetDROC^TM^) including ventrodorsal and lateral projections, demonstrated twin fetuses in all three cases (Figure 2). Two cases showed posterior fetal presentation, while one case had anterior presentation. Based on the imaging findings, relative fetal oversize, prolonged labor and the possibility of primiparity in the females were considered likely contributing factors to the observed dystocia. Gestational age and pregnancy duration were estimated using radiographic determination of fetal head position within the pelvic cavity, abdominal palpation, and in-house biometric data derived from day-old neonates obtained from prior cesarean section deliveries.

### Preparation of patient

Considering the small size of patients, manual fetal manipulation and obstetrical maneuvers were considered impractical. Animals were prepared for surgery, hairs were clipped followed by scrubbing and disinfection of surgical site with povidone iodine scrub (7.5%) followed by isopropyl alcohol.

### Anesthetic considerations

The intramuscular administration of midazolam (0.3 mg/kg) and butorphanol (0.15 mg/kg) was preferred as preanesthetic (11). Ketamine was avoided because it can cross the placenta, enter the fetal circulation, and may have neurotoxic effects on the developing fetal brain. General anesthesia was induced using a gas anesthesia machine delivering isoflurane (0.5%−4%) in oxygen via an anesthesia face mask. Once adequate muscle relaxation was achieved and the animal reached a surgical plane of anesthesia, it was placed in dorsal recumbency on the operating table. An appropriate surgical plane of anesthesia was confirmed by the absence of palpebral and interdigital reflexes. A 24-gauge intravenous catheter was placed in the brachial vein to allow rapid access for emergency drugs and fluid administration. Consistent with the established protocol, no supplemental ketamine was given after removal of the fetus.

**Figure 3:**
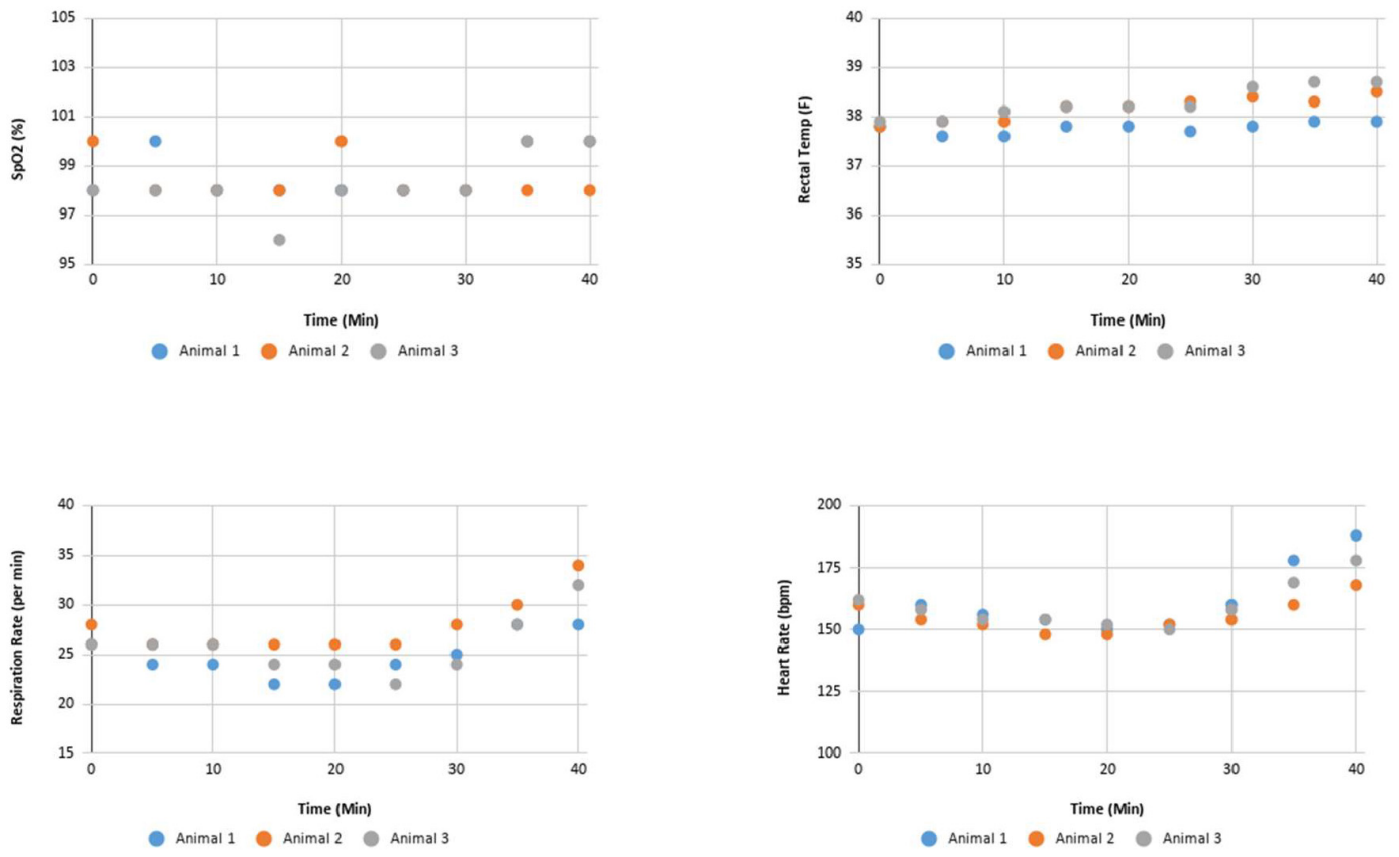
Continuous monitoring of vital parameters.

Amoxicillin –clavulanate (12.5 mg/kg) was administered for perioperative antibiotic coverage through intravenous canula. Fluid therapy with Ringer's lactate was maintained throughout the procedure using a syringe pump at a rate of 5 ml/kg/h. Physiological parameters—including heart rate, electrocardiogram (ECG), rectal temperature, oxygen saturation (SpO_2_), and respiratory rate—were continuously monitored using a multiparameter monitor during the surgery and remained within normal physiological limits throughout the procedure (Figure 3).

### Surgical procedure

**Figure 4:**
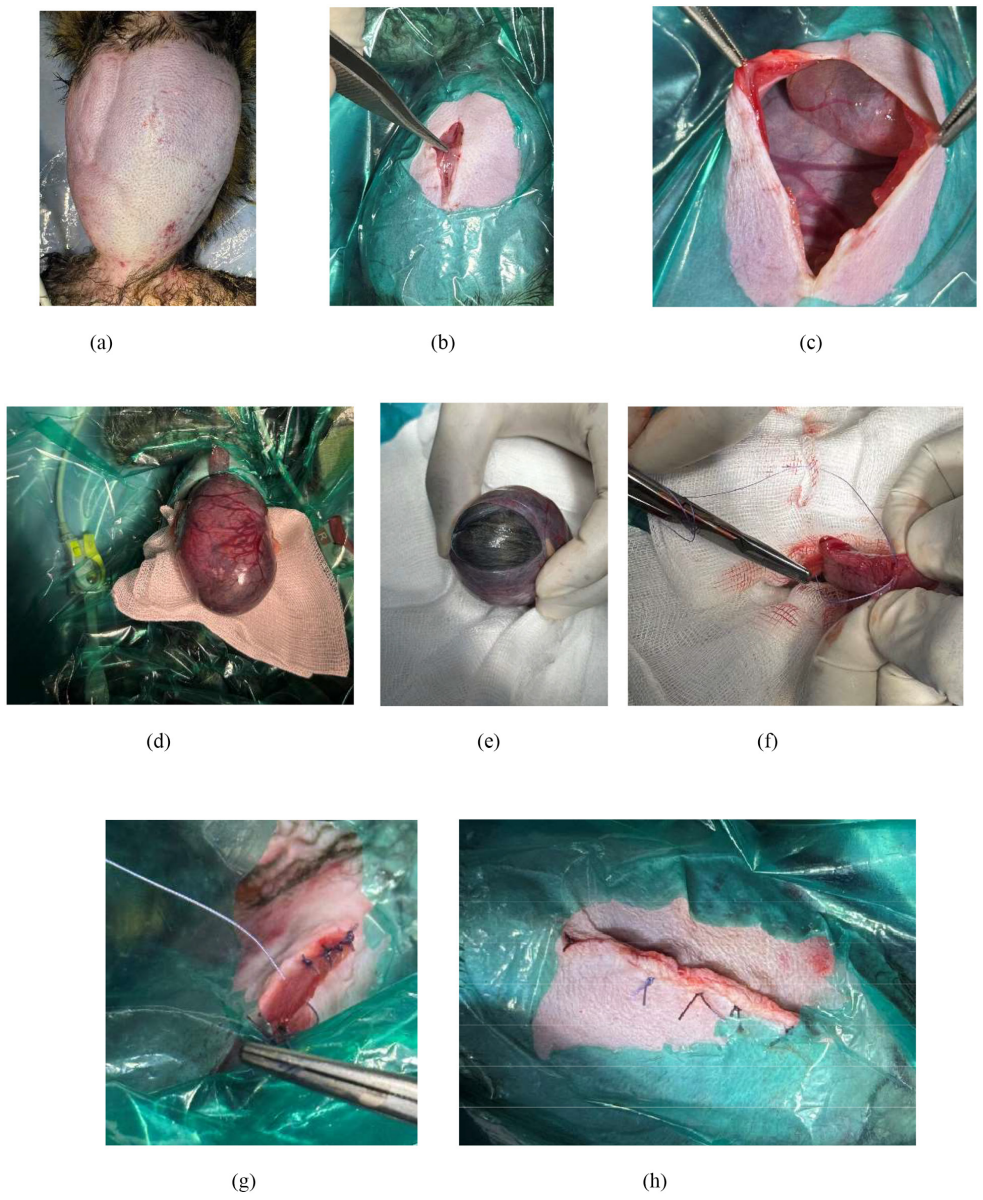
Illustration of the step-by-step surgical procedure **(a)** Preparation of surgical site **(b)** Incision **(c)** Access to the peritoneal cavity **(d)** Gravid uterus **(e)** Retrieval of fetus **(f)** Uterine incision closing **(g)** Suturing of abdominal musculature **(h)** Suturing of Skin incision.

Under aseptic conditions, a laparotomy was performed through a midline incision along the linea alba Figure 4a. Animals were maintained in dorsal recumbency, and a 5 cm straight midline incision was made through the linea alba Figure 4b. The abdominal cavity was carefully entered Figure 4c. The urinary bladder was manually emptied to improve surgical access. The gravid uterus was gently exteriorized, and a 2–3 cm transverse incision was made on uterine body at a relatively less vascular region a Figure 4d to facilitate removal of the fetuses and placentas Figure 4e. The umbilical cord of each fetus was clamped at two points and transected between the clamps.

The uterus was closed using Polyglactin 910 3–0 in Cushing's and Lembert's suture patterns, and the integrity of the uterine closure was carefully checked for leakage Figure 4f. The peritoneum and abdominal musculature were sutured with Polyglactin 910 3–0 using a continuous lockstitch pattern Figure 4g. Skin closure was performed using Nylon 3–0 in a horizontal mattress pattern Figure 4h. The surgical procedure lasted approximately 30 min from the initial skin incision to completion of skin closure, followed by an additional 10 min for recovery from anesthesia.

The animals recovered smoothly from anesthesia. Postoperative management included oral administration of amoxicillin–clavulanate for seven days, along with tolfenamic acid (4 mg/kg) administered intramuscularly and tramadol (2 mg/kg) administered subcutaneously for 3 days for analgesia and anti-inflammatory support. The surgical wounds healed without complications. Following treatment, the females were housed in isolated enclosures for 3 weeks for close monitoring before being returned to their respective enclosures.

### Neonatal care

Immediately after delivery, the neonates were provided supportive care, including clearing the nostrils and gently drying and warming them with a sterile towel. Each of the three females delivered twin offspring, resulting in a total of six neonates, comprising four females and two males. The average body weight of the neonates ranged between 29 and 32 g. Following initial stabilization, the neonates were transferred to a brooder for continued monitoring and care. All neonates were successfully hand reared. Biometric morphology was performed for all newborns and is presented in Table 3. 3 months of follow-up during hand-rearing revealed good survival rates and consistent weight gain in all neonates. A progressive increase in body weight was observed in all infants during the study period. Individual body weights at 1 month of age were 56, 50, 62, 50, 51, and 54 gms; at 2 months of age, 77, 74, 86, 64, 69, and 77 gms; and at 3 months of age, 129, 115, 125, 130, 118, and 120 gms. Throughout the observation period, all newborns remained physiologically normal and maintained optimal health status. All neonates survived the hand-rearing period and are scheduled for release into a larger permanent enclosure.

**Table 3:** Biometry parameters of black tufted marmosets.

Maternal body weights (gm)	Sex	Newborn weight (gm)	Tail length (cm)	Body length (cm)	Height at shoulder^1^ (cm)	Head width^2^ (cm)	Head length^3^ (cm)
400	Female	29	9.8	7.7	3.8	1.9	3.1
	Female	30	9.5	7.9	3.5	2.1	2.9
430	Male	30	10.0	8.1	3.4	1.8	3.0
	Female	31	9.2	7.8	3.7	2.0	2.8
450	Male	29	8.8	8.0	3.6	1.9	3.1
	Female	32	9.0	7.8	3.4	2.0	3.1

^**1**^Height at shoulder: The vertical distance from the tip of the middle digit of the forelimb to the highest point of the scapular region.

^**2**^Head width: The maximum transverse distance across the head measured between the outermost lateral points of the head.

^**3**^Head length: The linear distance from the most anterior point of the head to the most posterior point of the head.

## Discussion

Cesarean section in marmosets is generally described as a straightforward and well-tolerated procedure, and the surgical approach used in this study was in accordance with techniques reported in the literature. The anesthetic protocol followed established methods described in earlier publications in which inhalation mask was used for induction of anesthesia. This approach proved to be safe and effective in the animals included in this series. Infants require immediate care and close monitoring by skilled keepers. If hand-reared, they should be fed every 2 h using Nan Pro^TM^ Stage 1 milk formula. The infant's weight and activity level will be used to determine whether supplemental nutrition is needed (2, 12).

The decision to perform a cesarean section was guided primarily by animal welfare considerations and the practical limitations of attempting obstetric manipulations in such small-bodied primates.

Building on our previous single-animal study, this study established a comprehensive and reliable protocol, this report outlines a practical and reproducible approach that yielded favourable outcomes in the cases described, while acknowledging the limitation of the small sample size.

There is a notable paucity of data regarding neonatal morphometry and radiographic measurements of fetuses at full term, particularly in the immediate pre-parturition period, to reliably assess fetal maturity. While neonatal morphometric reference ranges may aid in identifying underdeveloped or oversized fetuses, the available data remain limited. Therefore, we propose to opportunistically collect and establish such reference data as cases present, without undertaking any additional or invasive handling of animals solely for research purposes.

In conclusion, this case series demonstrates cesarean section as a feasible and life-saving intervention in captive marmosets when dystocia or fetal distress is suspected. Prompt clinical assessment, timely surgical decision-making, appropriate anesthetic management, and careful surgical handling are key factors contributing to favorable maternal recovery and neonatal survival.

## Data Availability

The original contributions presented in the study are included in the article/supplementary material, further inquiries can be directed to the corresponding author.
